# Tricyclo­[6.2.1.0^2,7^]undeca-4,9-diene-3,6-dione

**DOI:** 10.1107/S1600536808030249

**Published:** 2008-09-24

**Authors:** Chongchen Wang

**Affiliations:** a School of Environment and Energy Engineering, Beijing University of Civil Engineering and Architecture, 100044 Beijing, People’s Republic of China

## Abstract

The title compound, C_11_H_10_O_2_, crystallizes with two independent mol­ecules in the asymmetric unit. In one mol­ecule, the dihedral angle between the mean planes of the C—C=C—C group of the diene unit and essentially planar cyclo­hexene ring is 51.07 (9)°, while in the other mol­ecule it is 54.49 (12)°. In the crystal structure, weak inter­molecular C—H⋯O inter­actions link the mol­ecules into columns along the *b* axis.

## Related literature

For background information, see: Ito *et al.* (2007 ▶); Mgani *et al.* (1995 ▶).
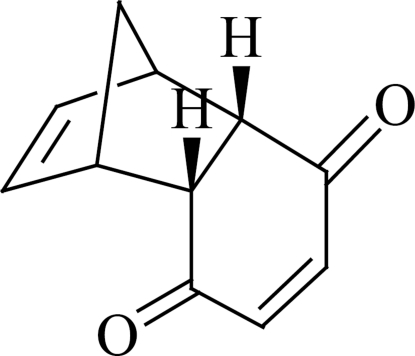


## Experimental

### 

#### Crystal data

C_11_H_10_O_2_
*M* *_r_* = 174.19
Monoclinic, 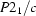

*a* = 15.649 (3) Å
*b* = 6.5399 (13) Å
*c* = 21.448 (7) Å
β = 125.05 (2)°
*V* = 1797.0 (8) Å^3^
*Z* = 8
Mo *K*α radiation
μ = 0.09 mm^−1^
*T* = 293 (2) K
0.52 × 0.12 × 0.11 mm

#### Data collection

Rigaku R-AXIS RAPID IP area-detector diffractometer
Absorption correction: multi-scan (*ABSCOR*; Higashi, 1995 ▶) *T*_min_ = 0.956, *T*_max_ = 0.990
6936 measured reflections
4119 independent reflections
2664 reflections with *I* > 2σ(*I*)
*R*_int_ = 0.036

#### Refinement

*R*[*F* ^2^ > 2σ(*F* ^2^)] = 0.066
*wR*(*F* ^2^) = 0.210
*S* = 1.03
4119 reflections
251 parameters
H-atom parameters constrained
Δρ_max_ = 0.22 e Å^−3^
Δρ_min_ = −0.21 e Å^−3^

### 

Data collection: *RAPID-AUTO* (Rigaku, 2001 ▶); cell refinement: *RAPID-AUTO*; data reduction: *RAPID-AUTO*; program(s) used to solve structure: *SHELXS97* (Sheldrick, 2008 ▶); program(s) used to refine structure: *SHELXL97* (Sheldrick, 2008 ▶); molecular graphics: *SHELXTL* (Sheldrick, 2008 ▶) and *PLATON* (Spek, 2003 ▶); software used to prepare material for publication: *SHELXTL*.

## Supplementary Material

Crystal structure: contains datablocks global, I. DOI: 10.1107/S1600536808030249/lh2695sup1.cif

Structure factors: contains datablocks I. DOI: 10.1107/S1600536808030249/lh2695Isup2.hkl

Additional supplementary materials:  crystallographic information; 3D view; checkCIF report

## Figures and Tables

**Table 1:** Hydrogen-bond geometry (Å, °)

*D*—H⋯*A*	*D*—H	H⋯*A*	*D*⋯*A*	*D*—H⋯*A*
C2—H2*A*⋯O4^i^	0.93	2.58	3.502 (3)	172
C6—H6*A*⋯O2^ii^	0.98	2.53	3.446 (3)	155
C11—H11*A*⋯O3^iii^	0.93	2.58	3.335 (4)	138
C16—H16*A*⋯O1^i^	0.98	2.59	3.416 (3)	142
C17—H17*A*⋯O4^iv^	0.98	2.51	3.319 (3)	140

Symmetry codes: (i) 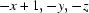; (ii) 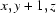; (iii) 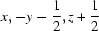; (iv) 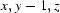.

**Fig. 1.**
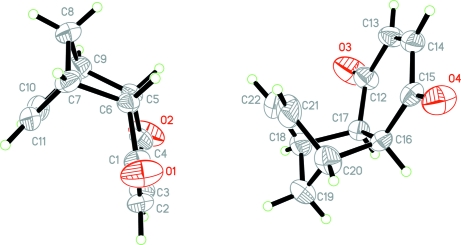
The asymmetric unit of the title compound, showing atoms labelling. Displacement ellipoids are drawn at the 50% probability level.

**Fig. 2.**
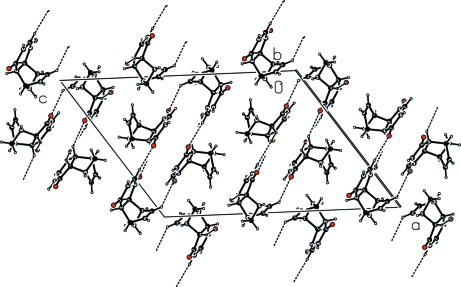
Packing diagram.

## supplementary crystallographic information

### Comment

The title compound, tricyclo[6.2.1.0^2,7^]undeca-4,9-diene-3,6 -dione formed by the cycloaddition between cyclopentadiene and *p*-benzoquinone has been investigated widely ( Ito *et al.* 2007; Mgani, *et al.* 1995). One of the unique aspect of the title compound is its high molecular symmetry, which allows for facile selective reactions at one or both carbonyl groups by means of classical and non-classical reagents. Another feature to be considered is its cage-like framework, which forces functional groups into close spatial proximity, facilitating subsequent reactions (Ito *et al.* 2007).

The title compound crystallizes with two independent molecules in the asymmetric unit, as shown in Fig.1. In one molecule, the dihedral angle between the mean planes of C-C=C-C group of the diene unit and essentially planar cyclohexene ring is [C1-C6] 51.07 (9)° while in the other [C12-C17] it is 54.49 (12)°. In the crystal structure, weak intermolecular C—H···O interactions link the molecules into columns along the b-axis (Fig .2).

### Experimental

Tricyclo[6.2.1.0^2,7^]undeca-4,9-diene-3,6-dione was obtained by the method described in the literature (Ito *et al.* 2007). The crystals of the title compound was recrystallized from hexane under low temperature (273.15 K).

### Refinement

All H atoms were fixed geometrically, with C—H distances of 0.93–98 Å with a mixture of treatments for the isotropic displacement parameters. In most case the isotropic displacement parameters were refined but in a few cases the value of U_iso_(H) = 1.2U_eq_(C).

### Crystal data

**Table:**

C_11_H_10_O_2_	*F*(000) = 736
*M**_r_* = 174.19	*D*_x_ = 1.288 Mg m^−^^3^
Monoclinic, *P*2_1_/*c*	Mo *K*α radiation, λ = 0.71073 Å
Hall symbol: -P 2ybc	Cell parameters from 16732 reflections
*a* = 15.649 (3) Å	θ = 1.6–27.5°
*b* = 6.5399 (13) Å	µ = 0.09 mm^−^^1^
*c* = 21.448 (7) Å	*T* = 293 K
β = 125.05 (2)°	Needle, dark brown
*V* = 1797.0 (8) Å^3^	0.52 × 0.12 × 0.11 mm
*Z* = 8	

### Data collection

**Table:**

Rigaku R-AXIS RAPID IP area-detector diffractometer	4119 independent reflections
Radiation source: Rotating Anode	2664 reflections with *I* > 2σ(*I*)
graphite	*R*_int_ = 0.036
ω oscillation scans	θ_max_ = 27.5°, θ_min_ = 1.6°
Absorption correction: multi-scan (ABSCOR; Higashi, 1995)	*h* = −20→20
*T*_min_ = 0.956, *T*_max_ = 0.990	*k* = −8→8
6936 measured reflections	*l* = −27→27

### Refinement

**Table:**

Refinement on *F*^2^	Primary atom site location: structure-invariant direct methods
Least-squares matrix: full	Secondary atom site location: difference Fourier map
*R*[*F*^2^ > 2σ(*F*^2^)] = 0.066	Hydrogen site location: inferred from neighbouring sites
*wR*(*F*^2^) = 0.210	H-atom parameters constrained
*S* = 1.03	*w* = 1/[σ^2^(*F*_o_^2^) + (0.118*P*)^2^ + 0.1983*P*] where *P* = (*F*_o_^2^ + 2*F*_c_^2^)/3
4119 reflections	(Δ/σ)_max_ < 0.001
251 parameters	Δρ_max_ = 0.22 e Å^−^^3^
0 restraints	Δρ_min_ = −0.21 e Å^−^^3^

### Special details

**Table:**

Geometry. All e.s.d.'s (except the e.s.d. in the dihedral angle between two l.s. planes) are estimated using the full covariance matrix. The cell e.s.d.'s are taken into account individually in the estimation of e.s.d.'s in distances, angles and torsion angles; correlations between e.s.d.'s in cell parameters are only used when they are defined by crystal symmetry. An approximate (isotropic) treatment of cell e.s.d.'s is used for estimating e.s.d.'s involving l.s. planes.
Refinement. Refinement of *F*^2^ against ALL reflections. The weighted *R*-factor *wR* and goodness of fit *S* are based on *F*^2^, conventional *R*-factors *R* are based on *F*, with *F* set to zero for negative *F*^2^. The threshold expression of *F*^2^ > σ(*F*^2^) is used only for calculating *R*-factors(gt) *etc*. and is not relevant to the choice of reflections for refinement. *R*-factors based on *F*^2^ are statistically about twice as large as those based on *F*, and *R*- factors based on ALL data will be even larger.

### Fractional atomic coordinates and isotropic or equivalent isotropic displacement parameters (Å^2^)

**Table:**

	*x*	*y*	*z*	*U*_iso_*/*U*_eq_	
O1	0.29192 (15)	0.0348 (3)	0.02418 (12)	0.0946 (7)	
O2	0.1016 (2)	−0.5875 (3)	−0.16845 (10)	0.1029 (8)	
C1	0.23966 (16)	−0.1048 (3)	−0.01874 (12)	0.0551 (5)	
C2	0.28081 (17)	−0.3137 (4)	−0.00061 (13)	0.0596 (6)	
H2A	0.3425	−0.3397	0.0467	0.074 (7)*	
C3	0.23343 (19)	−0.4665 (4)	−0.04914 (13)	0.0616 (6)	
H3A	0.2631	−0.5960	−0.0342	0.080 (8)*	
C4	0.13635 (19)	−0.4419 (4)	−0.12469 (12)	0.0581 (6)	
C5	0.07944 (16)	−0.2423 (3)	−0.14694 (10)	0.0490 (5)	
H5A	0.0655	−0.1990	−0.1957	0.067 (7)*	
C6	0.13399 (15)	−0.0649 (3)	−0.08969 (11)	0.0478 (5)	
H6A	0.1391	0.0514	−0.1162	0.066 (7)*	
C7	0.05338 (18)	−0.0092 (4)	−0.07124 (13)	0.0638 (7)	
H7A	0.0633	0.1236	−0.0468	0.082 (8)*	
C8	−0.04733 (19)	−0.0353 (5)	−0.15063 (15)	0.0781 (8)	
H8A	−0.1095	−0.0148	−0.1516	0.111 (11)*	
H8B	−0.0497	0.0498	−0.1886	0.082 (8)*	
C9	−0.02716 (19)	−0.2589 (5)	−0.15655 (14)	0.0706 (7)	
H9A	−0.0828	−0.3311	−0.2022	0.096 (9)*	
C10	−0.0019 (2)	−0.3395 (5)	−0.08217 (17)	0.0799 (8)	
H10A	−0.0175	−0.4691	−0.0735	0.096*	
C11	0.0467 (2)	−0.1914 (5)	−0.03133 (15)	0.0760 (8)	
H11A	0.0721	−0.1986	0.0199	0.091*	
O3	0.2497 (2)	−0.2686 (4)	−0.34967 (12)	0.1181 (10)	
O4	0.48892 (15)	0.3614 (3)	−0.18269 (12)	0.0870 (6)	
C12	0.3043 (2)	−0.1294 (4)	−0.30817 (13)	0.0652 (6)	
C13	0.28648 (19)	0.0779 (4)	−0.33792 (13)	0.0656 (6)	
H13A	0.2323	0.1012	−0.3886	0.092 (9)*	
C14	0.34485 (18)	0.2354 (4)	−0.29597 (14)	0.0620 (6)	
H14A	0.3289	0.3645	−0.3182	0.082 (9)*	
C15	0.43306 (16)	0.2141 (3)	−0.21624 (13)	0.0541 (5)	
C16	0.45436 (15)	0.0118 (3)	−0.17730 (11)	0.0504 (5)	
H16A	0.5278	−0.0221	−0.1540	0.050 (6)*	
C17	0.38776 (16)	−0.1714 (3)	−0.22614 (11)	0.0511 (5)	
H17A	0.4348	−0.2753	−0.2236	0.066 (7)*	
C18	0.34259 (19)	−0.2549 (4)	−0.18255 (14)	0.0665 (7)	
H18A	0.3164	−0.3956	−0.1948	0.070 (7)*	
C19	0.4353 (2)	−0.2168 (5)	−0.10083 (14)	0.0796 (8)	
H19A	0.4984	−0.2847	−0.0886	0.083 (9)*	
H19B	0.4212	−0.2508	−0.0636	0.129 (13)*	
C20	0.4363 (2)	0.0123 (5)	−0.11275 (13)	0.0755 (8)	
H20A	0.4863	0.0923	−0.0673	0.085 (8)*	
C21	0.3220 (3)	0.0601 (6)	−0.15010 (19)	0.0928 (10)	
H21A	0.2952	0.1807	−0.1449	0.111*	
C22	0.2675 (2)	−0.0992 (5)	−0.19120 (19)	0.0837 (9)	
H22A	0.1953	−0.1120	−0.2203	0.100*	

### Atomic displacement parameters (Å^2^)

**Table:**

	*U*^11^	*U*^22^	*U*^33^	*U*^12^	*U*^13^	*U*^23^
O1	0.0699 (12)	0.0600 (11)	0.0883 (13)	−0.0093 (9)	0.0071 (10)	−0.0161 (10)
O2	0.152 (2)	0.0603 (12)	0.0557 (11)	0.0017 (12)	0.0360 (12)	−0.0137 (9)
C1	0.0451 (11)	0.0487 (12)	0.0563 (12)	−0.0051 (9)	0.0203 (10)	−0.0048 (10)
C2	0.0436 (11)	0.0587 (14)	0.0559 (12)	0.0071 (10)	0.0165 (10)	0.0076 (11)
C3	0.0689 (14)	0.0461 (13)	0.0621 (13)	0.0107 (10)	0.0332 (12)	0.0048 (10)
C4	0.0789 (15)	0.0498 (13)	0.0433 (10)	−0.0029 (10)	0.0337 (11)	−0.0023 (9)
C5	0.0531 (11)	0.0533 (12)	0.0338 (9)	−0.0004 (9)	0.0210 (8)	0.0037 (8)
C6	0.0473 (11)	0.0420 (11)	0.0495 (10)	0.0025 (8)	0.0252 (9)	0.0044 (9)
C7	0.0566 (13)	0.0709 (16)	0.0573 (12)	0.0184 (11)	0.0290 (11)	−0.0016 (11)
C8	0.0511 (14)	0.107 (2)	0.0661 (15)	0.0232 (14)	0.0278 (12)	0.0209 (15)
C9	0.0462 (12)	0.091 (2)	0.0531 (12)	−0.0129 (12)	0.0158 (10)	0.0009 (13)
C10	0.0608 (15)	0.099 (2)	0.0882 (19)	−0.0061 (14)	0.0478 (15)	0.0189 (17)
C11	0.0644 (15)	0.116 (2)	0.0589 (14)	0.0220 (15)	0.0421 (13)	0.0195 (15)
O3	0.140 (2)	0.0854 (15)	0.0597 (11)	−0.0461 (14)	0.0168 (12)	−0.0131 (10)
O4	0.0699 (11)	0.0565 (11)	0.0983 (14)	−0.0123 (9)	0.0271 (10)	−0.0112 (10)
C12	0.0673 (14)	0.0638 (16)	0.0458 (11)	−0.0136 (11)	0.0216 (11)	−0.0050 (10)
C13	0.0593 (13)	0.0700 (16)	0.0463 (12)	0.0002 (11)	0.0179 (10)	0.0078 (11)
C14	0.0592 (13)	0.0550 (14)	0.0658 (13)	0.0049 (10)	0.0323 (12)	0.0107 (11)
C15	0.0439 (11)	0.0511 (13)	0.0637 (13)	−0.0030 (9)	0.0288 (10)	−0.0090 (10)
C16	0.0380 (10)	0.0581 (13)	0.0476 (10)	0.0017 (8)	0.0201 (9)	−0.0036 (9)
C17	0.0529 (11)	0.0498 (12)	0.0485 (11)	0.0011 (9)	0.0279 (9)	−0.0009 (9)
C18	0.0641 (14)	0.0672 (16)	0.0671 (14)	−0.0060 (12)	0.0370 (12)	0.0097 (12)
C19	0.0854 (19)	0.099 (2)	0.0553 (14)	0.0059 (16)	0.0409 (14)	0.0162 (14)
C20	0.0889 (19)	0.089 (2)	0.0512 (13)	−0.0114 (15)	0.0418 (13)	−0.0153 (13)
C21	0.123 (3)	0.095 (2)	0.113 (2)	0.024 (2)	0.098 (2)	0.005 (2)
C22	0.0706 (17)	0.098 (2)	0.104 (2)	0.0038 (16)	0.0628 (17)	0.0162 (19)

### Geometric parameters (Å, °)

**Table:**

O1—C1	1.219 (3)	O3—C12	1.215 (3)
O2—C4	1.224 (3)	O4—C15	1.220 (3)
C1—C2	1.464 (3)	C12—C13	1.456 (4)
C1—C6	1.493 (3)	C12—C17	1.496 (3)
C2—C3	1.321 (3)	C13—C14	1.327 (3)
C2—H2A	0.9301	C13—H13A	0.9300
C3—C4	1.460 (3)	C14—C15	1.462 (3)
C3—H3A	0.9299	C14—H14A	0.9300
C4—C5	1.495 (3)	C15—C16	1.497 (3)
C5—C6	1.542 (3)	C16—C17	1.538 (3)
C5—C9	1.563 (3)	C16—C20	1.564 (3)
C5—H5A	0.9799	C16—H16A	0.9800
C6—C7	1.567 (3)	C17—C18	1.559 (3)
C6—H6A	0.9800	C17—H17A	0.9800
C7—C11	1.505 (4)	C18—C22	1.483 (4)
C7—C8	1.526 (3)	C18—C19	1.523 (4)
C7—H7A	0.9800	C18—H18A	0.9800
C8—C9	1.517 (4)	C19—C20	1.522 (4)
C8—H8A	0.9702	C19—H19A	0.9700
C8—H8B	0.9700	C19—H19B	0.9700
C9—C10	1.502 (4)	C20—C21	1.514 (4)
C9—H9A	0.9801	C20—H20A	0.9801
C10—C11	1.324 (4)	C21—C22	1.314 (4)
C10—H10A	0.9300	C21—H21A	0.9300
C11—H11A	0.9300	C22—H22A	0.9300
			
O1—C1—C2	119.8 (2)	O3—C12—C13	120.0 (2)
O1—C1—C6	120.6 (2)	O3—C12—C17	119.7 (2)
C2—C1—C6	119.63 (18)	C13—C12—C17	120.3 (2)
C3—C2—C1	122.3 (2)	C14—C13—C12	122.8 (2)
C3—C2—H2A	118.8	C14—C13—H13A	118.4
C1—C2—H2A	118.9	C12—C13—H13A	118.8
C2—C3—C4	123.1 (2)	C13—C14—C15	122.6 (2)
C2—C3—H3A	118.3	C13—C14—H14A	118.7
C4—C3—H3A	118.6	C15—C14—H14A	118.7
O2—C4—C3	119.1 (2)	O4—C15—C14	119.4 (2)
O2—C4—C5	120.9 (2)	O4—C15—C16	121.0 (2)
C3—C4—C5	120.00 (19)	C14—C15—C16	119.67 (19)
C4—C5—C6	116.52 (17)	C15—C16—C17	117.52 (17)
C4—C5—C9	112.11 (19)	C15—C16—C20	113.36 (19)
C6—C5—C9	102.60 (18)	C17—C16—C20	102.03 (18)
C4—C5—H5A	108.6	C15—C16—H16A	107.7
C6—C5—H5A	108.3	C17—C16—H16A	108.0
C9—C5—H5A	108.3	C20—C16—H16A	107.8
C1—C6—C5	117.58 (17)	C12—C17—C16	116.78 (19)
C1—C6—C7	111.38 (18)	C12—C17—C18	112.29 (19)
C5—C6—C7	102.61 (17)	C16—C17—C18	103.15 (17)
C1—C6—H6A	108.4	C12—C17—H17A	108.2
C5—C6—H6A	108.3	C16—C17—H17A	108.0
C7—C6—H6A	108.1	C18—C17—H17A	108.0
C11—C7—C8	100.5 (2)	C22—C18—C19	101.2 (2)
C11—C7—C6	106.78 (19)	C22—C18—C17	106.5 (2)
C8—C7—C6	99.07 (18)	C19—C18—C17	99.93 (19)
C11—C7—H7A	116.0	C22—C18—H18A	115.5
C8—C7—H7A	116.2	C19—C18—H18A	115.9
C6—C7—H7A	116.0	C17—C18—H18A	115.6
C9—C8—C7	94.01 (19)	C20—C19—C18	93.6 (2)
C9—C8—H8A	112.7	C20—C19—H19A	112.9
C7—C8—H8A	112.9	C18—C19—H19A	112.7
C9—C8—H8B	113.1	C20—C19—H19B	113.1
C7—C8—H8B	112.9	C18—C19—H19B	113.2
H8A—C8—H8B	110.4	H19A—C19—H19B	110.5
C10—C9—C8	100.9 (2)	C21—C20—C19	99.8 (3)
C10—C9—C5	105.94 (19)	C21—C20—C16	106.9 (2)
C8—C9—C5	100.4 (2)	C19—C20—C16	99.8 (2)
C10—C9—H9A	115.7	C21—C20—H20A	116.2
C8—C9—H9A	116.1	C19—C20—H20A	116.0
C5—C9—H9A	115.7	C16—C20—H20A	115.8
C11—C10—C9	107.1 (3)	C22—C21—C20	107.8 (3)
C11—C10—H10A	126.4	C22—C21—H21A	126.1
C9—C10—H10A	126.4	C20—C21—H21A	126.1
C10—C11—C7	107.8 (2)	C21—C22—C18	107.4 (3)
C10—C11—H11A	126.1	C21—C22—H22A	126.3
C7—C11—H11A	126.1	C18—C22—H22A	126.3
			
O1—C1—C2—C3	−171.7 (3)	O3—C12—C13—C14	−178.5 (3)
C6—C1—C2—C3	8.3 (4)	C17—C12—C13—C14	3.1 (4)
C1—C2—C3—C4	−0.1 (4)	C12—C13—C14—C15	0.7 (4)
C2—C3—C4—O2	174.5 (3)	C13—C14—C15—O4	173.9 (3)
C2—C3—C4—C5	−7.1 (4)	C13—C14—C15—C16	−5.3 (4)
O2—C4—C5—C6	−175.9 (2)	O4—C15—C16—C17	−173.3 (2)
C3—C4—C5—C6	5.7 (3)	C14—C15—C16—C17	5.9 (3)
O2—C4—C5—C9	66.3 (3)	O4—C15—C16—C20	68.0 (3)
C3—C4—C5—C9	−112.1 (2)	C14—C15—C16—C20	−112.8 (2)
O1—C1—C6—C5	171.2 (2)	O3—C12—C17—C16	179.5 (3)
C2—C1—C6—C5	−8.9 (3)	C13—C12—C17—C16	−2.1 (3)
O1—C1—C6—C7	−70.9 (3)	O3—C12—C17—C18	−61.7 (3)
C2—C1—C6—C7	109.1 (2)	C13—C12—C17—C18	116.7 (3)
C4—C5—C6—C1	2.1 (3)	C15—C16—C17—C12	−2.3 (3)
C9—C5—C6—C1	124.9 (2)	C20—C16—C17—C12	122.3 (2)
C4—C5—C6—C7	−120.51 (19)	C15—C16—C17—C18	−125.9 (2)
C9—C5—C6—C7	2.3 (2)	C20—C16—C17—C18	−1.3 (2)
C1—C6—C7—C11	−61.5 (2)	C12—C17—C18—C22	−57.7 (3)
C5—C6—C7—C11	65.1 (2)	C16—C17—C18—C22	68.9 (2)
C1—C6—C7—C8	−165.4 (2)	C12—C17—C18—C19	−162.6 (2)
C5—C6—C7—C8	−38.8 (2)	C16—C17—C18—C19	−36.0 (2)
C11—C7—C8—C9	−49.1 (2)	C22—C18—C19—C20	−50.3 (2)
C6—C7—C8—C9	60.0 (2)	C17—C18—C19—C20	58.9 (2)
C7—C8—C9—C10	49.8 (2)	C18—C19—C20—C21	49.3 (2)
C7—C8—C9—C5	−58.9 (2)	C18—C19—C20—C16	−59.9 (2)
C4—C5—C9—C10	56.3 (3)	C15—C16—C20—C21	62.2 (3)
C6—C5—C9—C10	−69.4 (3)	C17—C16—C20—C21	−65.2 (3)
C4—C5—C9—C8	160.98 (18)	C15—C16—C20—C19	165.6 (2)
C6—C5—C9—C8	35.2 (2)	C17—C16—C20—C19	38.3 (2)
C8—C9—C10—C11	−33.6 (3)	C19—C20—C21—C22	−33.2 (3)
C5—C9—C10—C11	70.6 (3)	C16—C20—C21—C22	70.3 (3)
C9—C10—C11—C7	0.7 (3)	C20—C21—C22—C18	0.0 (3)
C8—C7—C11—C10	32.2 (3)	C19—C18—C22—C21	33.3 (3)
C6—C7—C11—C10	−70.7 (3)	C17—C18—C22—C21	−70.7 (3)

### Hydrogen-bond geometry (Å, °)

**Table:**

*D*—H···*A*	*D*—H	H···*A*	*D*···*A*	*D*—H···*A*
C2—H2A···O4^i^	0.93	2.58	3.502 (3)	172
C6—H6A···O2^ii^	0.98	2.53	3.446 (3)	155
C11—H11A···O3^iii^	0.93	2.58	3.335 (4)	138
C16—H16A···O1^i^	0.98	2.59	3.416 (3)	142
C17—H17A···O4^iv^	0.98	2.51	3.319 (3)	140

Symmetry codes: (i) −*x*+1, −*y*, −*z*; (ii) *x*, *y*+1, *z*; (iii) *x*, −*y*−1/2, *z*+1/2; (iv) *x*, *y*−1, *z*.
